# An Intelligent Traffic Surveillance System Using Integrated Wireless Sensor Network and Improved Phase Timing Optimization

**DOI:** 10.3390/s22093333

**Published:** 2022-04-27

**Authors:** Quadri Noorulhasan Naveed, Hamed Alqahtani, Riaz Ullah Khan, Sultan Almakdi, Mohammed Alshehri, Mohammed Aref Abdul Rasheed

**Affiliations:** 1 College of Computer Science, King Khalid University, Abha 61413, Saudi Arabia; hsqahtani@kku.edu.sa; 2 Yangtze Delta Region Institute (Huzhou), University of Electronic Science and Technology of China, Huzhou 313001, China; 3 Department of Computer Science, College of Computer Science and Information System, Najran University, Najran 55461, Saudi Arabia; saalmakdi@nu.edu.sa; 4 Department of MIS, College of Commerce & Business Administration, Dhofar University, Salalah 211, Oman; mohammed_aref@du.edu.om

**Keywords:** wireless sensor network (WSN), visual analytics, improved phase timing optimization (IPTO), traffic management system, computer vision

## Abstract

The transportation industry is crucial to the realization of a smart city. However, the current growth in vehicle numbers is not being matched by an increase in road capacity. Congestion may boost the number of accidents, harm economic growth, and result in higher gas emissions. Currently, traffic congestion is seen as a severe threat to urban life. Suffering as a result of increased car traffic, insufficient infrastructure, and inefficient traffic management has exceeded the tolerance limit. Since route decisions are typically made in a short amount of time, the visualization of the data must be presented in a highly conceivable way. Also, the data generated by the transportation system face difficulties in processing and sometimes lack effective usage in certain fields. Hence, to overcome the challenges in computer vision, a novel computer vision-based traffic management system is proposed by integrating a wireless sensor network (WSN) and visual analytics framework. This research aimed to analyze average message delivery, average latency, average access, average energy consumption, and network performance. Wireless sensors are used in the study to collect road metrics, quantify them, and then rank them for entry. For optimization of the traffic data, improved phase timing optimization (IPTO) was used. The whole experimentation was carried out in a virtual environment. It was observed from the experimental results that the proposed approach outperformed other existing approaches.

## 1. Introduction

Due to a variety of issues such as lighting fluctuations, camera calibration, and daytime settings, computer vision-based traffic vehicle monitoring remains a challenging aspect of any traffic surveillance system. As a result, performance needs are no longer left in research lab prototypes but are instead subjected to real-world difficulties. This desire renders the process of developing such a system extremely difficult, particularly when precision and speed are needed. Over the last decade, a lot of effort has gone into developing traffic surveillance systems which are designed to improve security by examining the on-road environments. In addition, a variety of sensing modalities, such as radar, lidar, and cameras, are becoming accessible for traffic monitoring. Simultaneously, computational power has risen considerably. Compared with wired configurations, wireless communication may save a lot of infrastructural development work, lessen the impact on existing traffic operation systems, and simplify maintenance tasks. New developing technologies not only make wireless networks more dependable but also make them more economical. The power, memory, and computing capabilities of WSN nodes are constrained. When it comes to battery power, sensor nodes in WSNs have to rely on limited and insufficient resources. One of the most important elements affecting the characteristics of a transceiver’s power consumption is the kind of antenna used. When the communication range is greater, more power is needed to send the messages. One of the primary research topics in WSN is the creation of a low-power communication system with an efficient antenna. Previously, WSN nodes have communicated by transmitting data in all areas using omni-directional antennas [1]. The broadcasting qualities of the omni-directional antenna restrict both bandwidth utilization and medium usage efficiency due to different variables such as “lower throughput”, “poor spatial reuse”, and “collision”. The use of directional antennas is recommended as a way to overcome the limitations of omni-directional antennas. In WSNs, directional antennas offer various benefits, including increased network capacity, greater transmission range, better spatial reuse, and reduced interference [2]. Directional antennas increase WSN performance by minimizing congestion and improving communication throughput range. Nevertheless, the performance of conventional systems is heavily reliant on their traffic object detectors, and it is worth noting that a traffic monitoring technique is becoming more trustworthy if the detector is sturdy [3].

Visual streams are used to depict facts in data visualization, translating diverse forms of data to suitable visualizations so that information analysis may be accomplished quickly. The benefit of data visualization is that it combines automation with human intelligence by incorporating human talents into an interactive visualization platform. The two key topics of data visualization are academic visualization and data visualization. Academic visualization depicts the spatial structures and evolvement of physiochemical attributes. The display of conceptual, unorganized, and high-dimensional data, such as corporate data, social media information, and textual information, is the subject of data visualization. Much effort has gone into establishing cost-effective and reliable traffic monitoring systems on a global scale. To increase traffic choices, one common method is to use computer vision to capture and evaluate relevant photos from existing urban video surveillance. Camera-based solutions such as this have modest infrastructural requirements and give vast aerial coverage, enabling traffic monitoring at multiple locations. The computerized system covers more ground than traditional traffic monitoring that relies on fixed-point sensors. Image processing methods are broadly utilized in a diversity of areas, such as aviation, healthcare, traffic control, and environmental object assessment [4].

The study of analytical reasoning helped by communicative visual interfaces is known as visual analytics, and it concentrates on producing human-computer practices and approaches for data processing, knowledge development, and problem resolution. It is an applied investigation subject which tries to provide practical solutions for a variety of application areas, including transportation. The best results are obtained when visual analytics investigators, who often lack subject experience, collaborate closely with domain experts. Unfortunately, although visual analytics investigators have performed extensively with transportation-related information and established a range of methodologies and instruments, which could be beneficial to transportation domain practitioners and researchers, such work has been restricted in the transportation field. Inadequate communication has two negative implications. Visual analytics investigators have a restricted awareness of the transportation domain’s challenges, needs, and constraints, which shall limit the utility and usability of the approaches they develop. The transportation community, on the other hand, is mostly unaware of the benefits that visual analytics may provide [5].

In terms of price, simplicity of installation and management, and better measurement capabilities, wireless magnetic sensor networks are an appropriate option for induction circuits for traffic control on motorways and at crossings. Wireless detection does have the ability to change the way traffic information is acquired by offering great geographic volume and precision observations. Inductive loop sensors, micro-loop probes, pneumatic road tubing, piezoelectric wires, and other weigh-in-motion monitors are used in many traditional traffic monitoring systems. These were selected due to their higher traffic detecting efficiency. To get the most out of these ITS solutions, large-scale implementation of traffic regulations on all major motorways and minor streets is required. As a result, real-time traffic data are necessary at these locations. Even though computer vision and visual analytics is a well-developed field of information technology, its identification, categorization, and tracking capabilities are not frequently used in daily life. As a result, we currently lack an automatic traffic control system for city roadways that can respond to changes in urban traffic in an automated manner [6].

### 1.1. Problem Statement

Many traffic visualization systems have been developed, but they are difficult to adapt for real-time traffic observation, assessment, and control in urban areas. The Social Internet of Vehicles (SIoV), which includes smart vehicles and networking units, is the primary operating premise of the Internet of Vehicle (IoV) platform. The majority of critical infrastructure is built-in, whereas others (mostly user-oriented and peripheral) could be inserted into the onboard diagnostics (OBD) connection being used as needed. For the smart city infrastructure’s proper functionality, constant and real-time communication is needed. Within the IoV infrastructures, there must be five different sorts of connections that must be made: “vehicle-to-infrastructure (V2I)”, “vehicle-to-vehicle (V2V)”, “vehicle-to-roadside unit (V2R)”, “vehicle-to-human (V2H)”, and “vehicle-to-sensors (V2S)”. No current visual methodologies give real-time traffic projections, which is critical for numerous activities including traffic signal modification and radio traffic broadcasts.

Hence, in this article, a computer vision-dependent traffic management methodology using an integrated wireless sensor network and visual analytics framework with improved phase timing optimization is proposed. The remaining sections of the paper are organized as follows: Section 2 emphasizes the literature evaluation. Section 3 describes the suggested methodology. Section 4 describes the performance and analysis of the proposed model. Section 5 demonstrates challenges and future directions in the field of IoV systems. Finally, Section 6 concludes the paper and demonstrates the idea of this research.

### 1.2. Key Points

Proposed Methodology: We have proposed an integrated wireless sensor network with a visual analytics framework and have employed improved phase timing optimization for a smart traffic management system. It was observed from the results that our model performed effectively in terms of accuracy and model fitting towards statistical data. The algorithmic structure of IPTO and the detailed proposed framework is given in Section 3.

Knowledge of challenges and future directions: this work highlights the current challenges in traffic management systems (e.g., security and privacy, reliability, interoperability, real-time communication, multi-model sensing, or heterogeneity) and also discusses the future directions for smart IoV environments. We demonstrate how artificial intelligence and blockchain-based solutions can contribute to building smart infrastructures and scientists should work on these emerging technologies. 

## 2. Related Works

Wang et al. [7] proposed a realistic strategy for actual-time traffic maintenance offloading in fog-dependent Internet of Vehicles (IoV) systems, intending to reduce the mean reaction time for incidents provided by vehicles. By partitioning the offloading optimization problem into two sub-problems and scheduling traffic flows amid distinct fog nodes, an approximation solution is devised to solve it. They will look into using automobiles outside of the communication ranges of “road side units (RSUs)” as fog nodes to offload loads for TMS in the future. As a result of this, it is tasked with processing all of the system’s communications, which might lead to excessive resource usage.

Yuan et al. [8] conducted a comprehensive review of 259 publications released in the last decades, as well as relevant performances before 2010, to better identify prospective research subjects and learn how to use relevant visual analytics approaches. They created taxonomy with three first-level classifications: methods before model construction, methods during model construction, and methods after model construction. Each class is further defined by a series of relevant analysis tasks, each of which is typified by a collection of current gained works. They also addressed and highlighted research problems as well as interesting future research prospects relevant to visual analytics.

Sumi and Ranga [9] suggested a smart traffic management approach for nations based on the IoT and the vehicular ad hoc network principles (VANET). Emergency vehicles are prioritized in the proposed approach for a smooth flow through traffic depending on the kind of occurrence. It directs ambulances to the smallest probable routes to their destination, and it also offers a way to identify and react to traffic signal hacking. In terms of congestion avoidance, travel duration, and energy consumption, our solution exceeds these suggestions for emergency vehicle systems.

Ning et al. [10] proposed a realistic strategy for minimizing traffic management services response time by permitting real-time content distribution in IoV systems depending on diverse network access. For large-scale IoV systems, they initially create a crowd sensing-dependent framework. Furthermore, to provide timely replies for traffic control, a cluster-based optimization framework is explored. They assume that the messages generated by vehicles are trustworthy. This may not always be the case, however, due to the possibility of false or inaccurate information being broadcast across the network to mislead other vehicles and traffic control systems.

Tsang et al. [11] developed a completely integrated method for traffic monitoring by combining high-definition intelligent cameras with wireless connectivity. This system will be known as a “computer vision-based roadside occupation surveillance system (CVROSS)”. Actual-time roadside traffic photos, such as photographs of loading and unloading operations, are acquired autonomously using a vision-based system. Decision assistance on roadside availability and vacancies could be analyzed using fuzzy logic and monitored for customers using the recorded information, improving the openness of roadside operations. To improve the visibility of roadside actions, further steps can be taken to examine, improve, and apply the CVROSS. CVROSS decreased traffic congestion and double-parking incidents by 41.2 percent and 33 percent, respectively. This technique cannot be used in other areas where there is double parking and busy roadside activities.

A systematic framework for constructing a network-level traffic congestion analytical tool that is ideal for traffic control and practical traffic management on roadways was offered by Gunda [12]. Detection of traffic spots, network congestion charts, input data quality analysis, traffic variation, and congestion performance evaluation are all key components. In addition, a novel pattern matching method was created to fill in the gaps in the data. This system, however, did not have a message scheduling mechanism to help with data transfer issues.

Lee et al. [13] introduced an interactive visual analytics system that uses vehicle detection information to support traffic congestion investigation, monitoring, and predictions. This visual analytics technology is made to permit customers to investigate the origins, routes, and intensity of traffic congestion. The congested circumstances of a town are depicted utilizing a volume-speed rivers visualization, which shows traffic levels and velocities at the same time. However, they found no evidence of enhanced performance as a consequence of the data in the experiments.

Riveiro et al. [14] proposed a visual analytics framework that supports: (1) multidimensional road traffic information analysis; (2) examination of normal behavioral models generated from information; (3) abnormal activity identification; and (4) abnormal incident explanations. The experts also identified several issues that need to be addressed, including the need to improve the assessment of observed abnormalities, as determining why the identified incidents are abnormal remains a challenge, and the limitations of the circular layout when a large number of attributes are chosen.

To solve the current restrictions, Nguyen et al. [15] suggested an extensible smart traffic management platform (STMP) centered on untrained deep learning methods. The STMP combines diverse big data streams, such as IoT, sensor systems, and social networks, to identify concept deflections, differentiate among recurring and non-recurring traffic occurrences, and perform brunt spreading, traffic flow predicting, and enhanced traffic regulation judgments. However, anticipating traffic flow with extreme changes that occur at a greater frequency has difficulties. When more traffic data (e.g., roadworks, accidents, and events) is utilized to train the DNN model, this issue should be addressed. Information from multiple sources, such as surveillance cameras, meteorological data, and other transportation-related data sets, will be fused in future research initiatives. Furthermore, to obtain platform adoption, the interpretability of artificial intelligence (AI) components, particularly those based on sophisticated approaches such as deep neural networks, should be investigated in the future.

Adu [16] suggested a novel architecture for carrying out multidimensional visualization and analytics on huge transportation information in a consistent way. The system stores information in a highly parallel dataset and uses the enormous computational capacity of “graphical processing units (GPUs)” to do real-time data analytics and renderings using a structured query language that interfaces with the underlying GPU databases. A front-end is meant to present query results on simplistic graphs and maps in near-real-time, allowing decision-makers to swiftly drill down into information. The technology is used to create apps that analyze large transportation databases with over 100 million rows. The technique created is capable of providing real-time visual upgrades for large datasets in less than 100 milliseconds, according to performance benchmarking trials. The proposed platform’s efficiency was also contrasted to CPU-based visual analytics tools such as Tableau and D3. As the paper also says, the created framework’s query response rates were around 10 times quicker than those of the two CPU platforms. This framework’s inability to handle non-structured data is a major flaw. It is assumed that the data are in tabular format and that data types such as video and images are not present.

A visual analytic system connected to the analysis of motion and transportation networks was reported by Lock et al. [17]. This system analyses the possible added value of fast, 2D, and 3D online visualization and data analytics modules in the study of large public transportation performance information. An innovative technique to showing such information is illustrated using a generalized framework visualization system. In a quick, interactive browser-based ecosystem, this system remembers almost a year’s benefit of public transportation reliability information to a great detailed degree. There may still be issues with the data itself when dealing with a large amount of data. As a result, real-time data may not always be accurate due to network and GPS problems. As a result of these concerns, operators might use “general transit feed specification (GTFS-RT) validation tools.” If GTFS-RT is widely used to monitor systems on a continuous rather than real-time basis, it will be easier to exchange knowledge and improve the tools for creating and validating data feeds.

De Souza, et al. [18] discussed traffic management system categorization, evaluation, difficulties, and prospects. A qualitative investigation was also conducted using the traffic management systems (TMS) mentioned. Lastly, they explained how they plan to enhance TMS efficacy and resilience to accomplish the desired level of precision and traffic monitoring, with significance on concentrating open complexities. Moreover, they have detected and discovered numerous feasible solutions. In addition to cloud computing’s inherent security issues, TMS also adds to the difficulty of securing a system.

Zhang, et al. [19] suggested a crowdsourcing-dependent traffic surveillance strategy, which allows transportation management to obtain road traffic information at road crossings in a time-saving, reliable, and confidential way. This approach’s main drawback is its inability to present huge datasets. They will use blockchain technology to assess the legitimacy of a crowdsourcing-based traffic surveillance situation in the future.

Rego et al. [20] suggested a newer control scheme depending upon the combination of “software defined networks (SDN)” and “Internet of Things (IoT)” in the nation’s surroundings. This system is activated when an emergency occurs and changes the regular and urgent traffic routes in urban areas to minimize the time it takes for emergency resources to arrive at the scene of an emergency. Studies reveal that the average time it takes emergency responders to arrive at an emergency site is decreased from 17 to 12 milliseconds. The proposed algorithm controls the resource requests and the route modification for helping the emergency service vehicle’s movement. This system’s inability to capture multidimensional perspectives of the data being shown is a major flaw.

Hashemi, et al. [21] presented a new trafficking network in real-time based on an end-to-end deep learning (E2EDL) technology. The suggested framework includes the network’s spatial and temporal l congestion profile correlations and links them with effective traffic management techniques. The E2EDL model is trained with a lab-generated data set that includes pairs of current traffic characteristics and effective traffic control strategies planned to deal with them. As a result of the system’s recommendation of routes to the same spot, however, congestion is created in different parts of town.

Ahmed, et al. [22] described a travel route suggestion procedure that can be used to suggest the best congestion-aware path in a network. Congestion indexes are calculated using both equipped and non-equipped automobiles, as well as driving distraction variables. This procedure is employed for designing smarter transportation systems to encounter traffic congestion issues. This system’s main drawback is that, in order to alleviate traffic congestion, it selects lengthy routes.

Jain, et al. [23] suggested vehicle social networks based on the VIoT in this research are made up of a larger number of sensors that wirelessly transfer data. The efficacy of traditional layered protocol solutions and existing cross-layer solutions for wireless systems is limited by the great heterogeneity in equipment capacities of things and quality of service (QoS) necessities for diverse uses. The system’s flaw is that it lacks a broadcast suppression mechanism, which reduces its efficiency, particularly in high-density scenarios.

Shelki, et al. [24] presented a framework for delivering adaptive traffic signal control and emergency service management in automobile ad-hoc networks by optimizing the acquisition, categorization, scheduling, and distribution of traffic data. The former provides for dynamic traffic signal regulation and traffic flow, and the latter permits emergency vehicles to pass at full speed. Sensor nodes in the proposed method analyze traffic data and share it with a “dynamic traffic management center (DTMC)”. It uses fuzzy logic to dynamically decide the road segment’s priority as critical, high, medium, or low. When enormous volumes of data are sent through networks, there is a larger risk of security vulnerabilities.

## 3. Proposed Work

**Figure 1:**
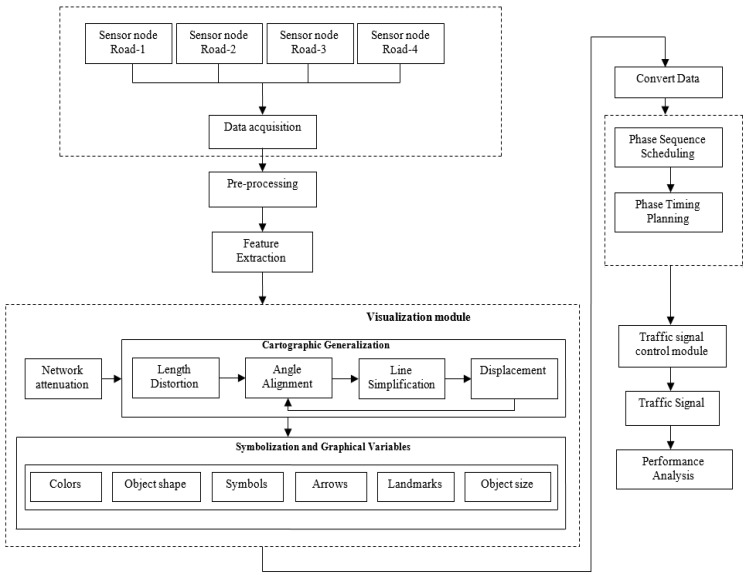
The schematic representation of the proposed method.

This section explains the flow of the proposed system. Currently, people have been processing various data to view, analyze, and transform the data to another form regularly. Automated vehicles, e-health care, population surveillance, and aerial surveying via satellite missions are just a few of the smart world’s applications. Visual analytics also offers several approaches for combining the advantages of humans and computers to digitize the globe. Visual analytics also provides a variety of graphical user interfaces, statistical reporting, interactive gadgets, and data management services to help the smart world develop. Furthermore, combining computer vision with visual analytics gives an amazing, cutting-edge solution for academics and engineers to share and assess inventions in the digitalized smart world, thereby contributing to a good environmental impact. Hence, in this paper, we have integrated wireless sensor network with visual analytics framework and have employed improved phase timing optimization for a smart traffic management system. The schematic representation of the proposed methodology is illustrated in Figure 1.

### 3.1. Data Acquisition

The raw data are collected and passed to the data preprocessing module during data collecting. Data can be obtained in a different format and from a variety of sources. This component must assimilate the necessary data, which will be sent in a unified format to the other modules. The sensor nodes provide the traffic information. The datasets utilized in this study are from the California Department of Transportation’s Performance Measurement System (Caltrans). This system is made up of about 39,000 Vehicle Identification Stations, which analyze vehicle flow and velocity and are located throughout the state’s highway system in all major urban regions [25].

### 3.2. Data Pre-Processing

Parameters such as kinds of vehicles, vehicle parking space requirements, and minimal traffic lane widths must all be set up before the suggested system can be operated. The Hong Kong Special Administrative Region Planning Department has developed rules that relate to these. As a result, the system may contrast the collected photos to templates in the database, allowing for more precise image and data processing in the future. Variations in the size of everything produced by non-equivalent distance from the vision equipment are neglected in the computing machine to make it easier to calculate parking gaps and accessible parking areas. In another sense, irrespective of its orientation in association to the vision equipment, each of the things shown in a case is presumed to have the same measurements in millimeters or pixels. Initial variables in the calculating procedure involve:

- The vision device’s total coverage is 640 × 480 pixels.
- Each truck must have 11 m of the controlled parking area.
- Each cargo van requires 7 m of controlled parking areas.
- Each private car has a 5 m-controlled parking place.
- The minimum width of traffic lanes is 6.75 m.
- All conceivable vehicles and things are represented as templates.
- A trust value for every pixel for every template, indicating how confident the discrepancy is.

The phase begins the first level of the timed loop, vision collection after the variables are supplied into the developed framework. The platform’s wireless vision sensors may then continuously and automatically capture photos from the roadway. Following that, the information is used for (i) noise minimization, (ii) vehicles and objects detection and matching, (iii) data filtering and refining, and (iv) data imputation and validation.

### 3.3. Noise Reduction

Noise reduction is among the most critical phases in the overall network flow. This is a technique for reducing noise from an image, which can affect both the visual qualities and the efficiency of future processing operations. There are various objects and signals, such as traffic indicators and directions in traffic streams, on the roadside, and in traffic lanes in this scenario (according to the simulation). Nevertheless, since these are presumably not related to vehicle and object detection and comparing, they shall harm matching findings and the effectiveness of following parking space calculations. Moreover, even identical vehicles, such as two individual cars in this condition, shall be of a similar design but varying in color. Image quality may be assessed using the signal-to-noise ratio (*SNR*). An imaging system’s sensitivity is commonly measured in terms of the *SNR*. When the signal is an optical intensity, it is defined as the ratio of the average signal value $\mu_{sig}$ to the standard deviation of the signal $\sigma_{sig}$, or as the square of this value when the signal and noise are seen as amplitudes.

(1) $$SNR=\frac{\mu_{sig}}{\sigma_{sig}}$$

It may guarantee that irrelevant items, signs, and indications are deleted from images before they are subsequently processed. It also eliminates color classification issues. Before noise mitigation, the image created from vision acquisitions was loaded with obstructions such as a highway sign, a traffic cone, and yellow circle marks. It was tough to distinguish and identify cars and goods as a result of all of this. Noise minimization was attained utilizing an “image mask” to prevent unrelated areas of the images, “color plane extraction” to transform the color picture to a binary picture in only black and white, and “basic morphology” to enhance the structure of binary objects in the picture, and also to modify the illumination. Following noise minimization, the indicator, traffic cone, and yellow circle marks are eliminated and only the individual vehicle stayed on the display with its structure displayed in white.

### 3.4. Vehicle and Object Recognition and Matching

The proposed system includes two typical matching methods: pattern matching and geometric matching. Pattern matching is the optimal solution if all of the things, which need to be identified and matched, have the same characteristics, as it compares every characteristic and color of an item from the templates and the observed images. All cars and items, meanwhile, are not created equal. Certain proprietors, for instance, may color a vehicle’s top or body. As a result, not every object has the same designs or colors. This could harm the detection and matching of vehicles and objects. As a consequence, geometric matching appears to be more appropriate for utilization in the proposed system to identify, detect, and compare various kinds of vehicles and objects depending on their forms, extents, and other important characteristics, and also to evaluate the image score values, when combined with noise. This can avoid an object from being misidentified and mismatched owing to different colors and patterns. When the image has been adequately collected and the noise has been removed, the procedure of identification and matching shall begin. Templates inserted throughout the system parameters set-up procedure are used since the cars and items must be recognized and matched. When an object is identified, a vehicle passes by, or a vehicle parks within the HD vision devices’ field of view, the devices will automatically record photos and contrast them to templates in the databases. Following identification, vehicles and objects could be allocated to a categorization.

### 3.5. Data Filtering and Refining

The content in real-world data is repetitive. Data filtering and refinement is the method of removing duplicates, contradictory information, and noise from information while protecting integrity. This is necessary to have reliable modeling results.

### 3.6. Data Imputation and Validation

Sensor failure can cause observations to be incorrect, ignored, or distorted. As a result, several preparatory tasks such as data imputation and data validation must be completed to effectively analyze the data. This process involves filling in incomplete data and assessing data performance and consistency.

### 3.7. Visualization Module

We present an approach that unifies several ways to visually suggest efficient paths to automate the visualization process. Several cartographic generalization methods, such as feature extraction, simplifying, schematization, and deformation of map objects, as well as the utilization of symbolization and graphical parameters, are used in this framework.

Visual analytics investigation is primarily focused on graphical and semantically complications, which lends itself to a more in-depth interaction of the cartography sector. To handle a large range of location dimensions, cartographers would have to invent several generalization approaches. Although cartographic generalization is far from fully known, let alone computerized, it is difficult to imagine a society more dedicated to the subject or one with greater factual expertise. By striving to tackle both geometrical and cognitive components of mapping difficulty, the cartographic strategy to generalization tends to differentiate itself among purely visual complexity strategies, such as the famous level-of-detail concept in graphic design. 

The following route visualization process is divided into two sections: firstly, cartographic generalization, and secondly, the utilization of symbolization and graphical parameters. These two components are developed to function together that can also be used separately. Depending on cognitive psychology investigation, both forms of visualizations incorporate a variety of operations. Significantly, certain operations aren’t applicable in all cases, while others will use a different approach based on the information. In the same way, certain operators are required for the visualization mechanism while others are discretionary. The architecture, such as the situations, may be dynamically enhanced by adding more visualization operations. The displacement of the length of road sections, such as alternate visualization methods developed as the portion of the model employing cartographic generalization, combines several ways depending on the context. The length of road sections is changed in the standard scenario depending on the perceived lengths of road sections. We handle other temporal aspects such as current traffic density as input for generalizing road segment length in the other traffic-related scenarios. The theory is that peoples’ perceptions of road length are influenced by the actual travel time required to complete the path [18].

### 3.8. Length Distortion

**Figure 2:**
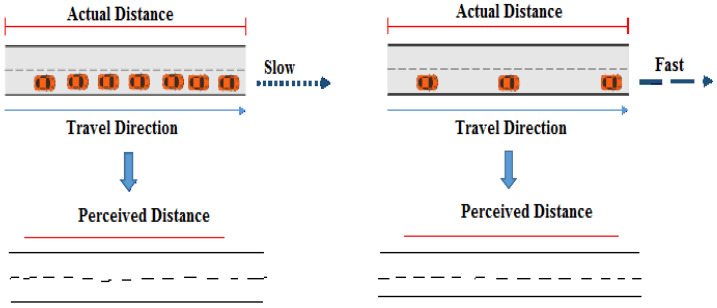
The actual length of a road segment as contrasted to the perceived length.

The premise that travelers evaluate road length based on actual travel time instead of the actual measurements of a road section motivated us to build an algorithm that dynamically alters the lengths of a particular line section. As said before, journey time is also a significant issue in spatial choice models. Figure 2 shows how, depending on traffic density, the perceived distance between two points along a path can differ from the actual metric distance. The perceived distance is not easily quantified in terms of metrics, unlike the metric distance between two places along a road section, since it is not a set score and changes based on individual variances in human perception of space and time.

We utilized the PUSH software [19], which contains an expanded option to scale objects by a component, to deform the lengths of a line segment. PUSH is a software application that was created to dynamically generalize cartographic items and, in specific, eliminate spatial conflicts through dislocation. It provides for a very flexible description of object behavior during dislocation by specifying numerous characteristics, such as the permissible degree of distortion or the minimal space needed by an object. Each object’s attributes could be customized separately. To determine the enlarged factor autonomously, we firstly determine the expected perceived length (*t*) of a road segment using the equation:

(2) $$plenght\left(t\right)=len\left(t\right).\frac{\theta dens\left(t\right)+\left(dens\left(t\right)-\theta dens\left(t\right)\right).w_{g}}{\theta dens\left(t\right)}$$

The estimations are dependent on the present traffic density *dens* (*t*), calculated for every individual road section as contrasted to the mean traffic density θdens(t) for the road section during a similar period. 

We also present a weight factor $w_{g}$, that permits to dynamically modify the intensity of the variation of the attained length value from the actual length value len (*t*), using

(3) $$w_{g}=\left\{0,\;2\right\}\;\in \;Q$$

This weighting factor allows for individual variances in observed road length to be addressed, as well as for traffic control to intervene if the strength of aberrations needs to be physically increased or decreased. The enlarge factor is then computed as the ratio of the estimated observed length of the line segment to its actual length:

(4) $$enlarge=\frac{plenght\left(t\right)}{len\left(t\right)}$$

Once the individual enlarges factors have been set to the line segments that need to be scaled, the global optimization finds a holistic solution that takes all of the other objects into consideration (i.e., relocating them suitably if necessary). The line segments of the generated polyline will be scaled based on the provided traffic volume estimates. That is, high-volume segments grow in size while low-density segments shrink in size. If the estimated traffic volume is equal to the mean volume for the road section at a given moment, the expand factor does not affect the segment’s depiction. Since the entire picture is optimized in one step, bigger scores for the aura specification for the matching line sections can be defined. This dislocation of map elements shall be extremely helpful in visually distinguishing between diverse pathways in a system.

### 3.9. Line Distortion

According to the earlier study, visualizing a road section as a straight line is related to a very smooth motion in space [26], and therefore with a high traffic flow. Roads with lesser traffic flow (or high traffic volume) may, on the other hand, be related to a less stable way of a line that alters from a straight-line representation. While there are numerous widely used methods for reducing the complexity of a line, there are only a few approaches that aim to deform a line automatically [27]. In this section, we use a method that artificially deforms the shape of a provided line section utilizing traffic-associated information as an input for distortion.

### 3.10. Improved Phase Timing Optimization (IPTO) Algorithm

To tackle the optimization problem, a genetic algorithm is used in this part. GAPTR stands for a genetic algorithm-based phase timing rescheduling technique. We encode the green time of four separate phases in GAPTR. The binary integer 0 or 1 represents each gene on the chromosome. Each chromosome reflects a unique attempt to time phase transitions. The fitness function is used to assess those who are at the top of their game. Better people are more restrained and have more opportunities than those who are less fortunate. The goal of GAPTR is to reduce the length of waiting for vehicles’ queues. As a result, in this paper, we use the goal function as the fitness value explicitly. We use traditional genetic methods for the choice, crossover, and mutation operators. For instance, in the selection process, a roulette-wheel technique is utilized, and in the crossover and mutation operations, multiple point crossover and mutation are used.

### 3.11. Algorithm for IPTO

The crossover operation appears to swap gene segments in phases, but this is not required in practice. To perform the crossover procedure, we can use a random binary vector of the same length as the chromosomes. The value of a component in the vectors indicates which parent the gene for the offspring’s corresponding location came from. If the value in the present location of the vector is 1, for instance, the gene value in the corresponding point of the offspring originates from parent 1, and if not, parent 2. Mutation operations with random vectors could be performed similarly. The main distinction is that the value of the component in the vector indicates whether or not the gene in the offspring at the appropriate site propagates as shown in Algorithm 1 below.
**Algorithm 1:** IPTO.  **Input:** sensor traffic data   **Output:** traffic management data   **if** this. Host is cluster head = TRUE then return   Send a message to cluster head (this. Messages)   **end if**   loc of roadside unit ← GETLOCOFNEARESTRSU   shortest Paths ← GETSHORTESTPATHSBASED Map   DelaysforPaths ← COMPUTEDELAYSFORPATHS   isUpBS ← GETISUPLOADBYBS   if isUpBS then return   UPLOADMESSAGESBYBS   **end if**   neighbors ← GETALLNEIGHBOURS   **for** i ← 0 To Length[neighbors] do   isMoving ← IS MOVING ON THE SHORTEST PATHS-S(neighbors[i].loc)   if isMoving == TRUE then   ncTurningPos ← COMPUTETURNINGPOS   ne TurningPos ← COMPUTEENTURNING POS   if this.cTurning Pos > threshold And   ncTurningPos > this.cTurning Pos Andneighbors[i].    estimated Delay < this.estimated Delay   **then**   SENDMESTONEIGHBOR   end if   if this.cTurning Pos < threshold then   if ncTurningPos−this.cTurning Pos > ϵ   then   SENDMESTONEIGHBOR   ElsencTurningPos−this.cTurningPos < ϵ   And neTurningPos > this.eTurningPos   SENDMESTONEIGHBOR   **end if**   **end if**   **end if** **end for**

## 4. Performance Analysis

### 4.1. Computational Environment Settings

To test the system’s performance, we used a city map. OpenStreetMap [28] was used to create this map. The tool OS-M2WKT [29] is used to transform OpenStreetMap XML files to WKT format. These files, which are in text format, are used to store map-related information. The motion of cars is based on the shortest path map, and the average arrival rate of traffic flow on every route section is determined depending on the vehicle motion historical documents. The simulation lasts 168 h, and the vehicle’s wireless communication range is 40 m. The message size ranges from 200 to 500 megabytes, and the message Time-To-Live (TTL) is 30 min. A vehicle’s velocity is between 20 and 60 km per hour, and communication costs $0.007 per megabyte. To get the average score of every performance metric, we run each simulation scenario 100 times. The subsequent four performance indicators are looked as the following subsections.

### 4.2. Average Delivery Ratio

The overall created messages are divided by the number of messages, which can be analyzed by TMS. To calculate the average delivery ratio, we must divide the total number of messages sent by a sender to a destination host in the system by the total number of messages that were received. The goal is to send as much information to its destination as possible.

### 4.3. Average Latency

The mean time it takes for TMS to receive a message from the time it is created. That means that the period between when the sender begins transmitting the message and when the recipient accepts the message is referred to as average delivery delay.

### 4.4. Average Communication Cost

The overall count of messages posted is divided by the total count of messages TMS has reacted to.

### 4.5. Access Ratio

The proportion of roadside unit (RSU) uploaded messages to base-station (BS)-uploaded messages.

**Figure 3:**
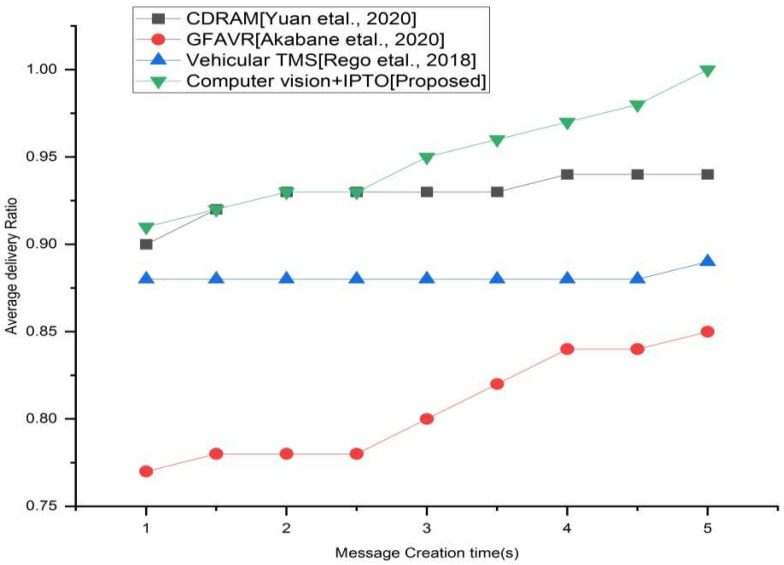
Average delivery ratio vs. different message interval for the existing and proposed method.

The performance of the suggested system is contrasted with the traditional systems in terms of these metrics. Depending on the city map, Figure 3 shows the average delivery ratios of the four methods (CDRAM, GFAVR, vehicular TMS, and suggested). The suggested system outperforms GFAVR and vehicular TMS in terms of performance. Here, the proposed technique has a greater delivery proportion than that of the existing approaches. That means the proposed approach has a proportion of 0.912 more than the existing approaches CDRAM (0.90), GFAVR (0.78), vehicular TMS (0.88) in the first second of message creation time. In this work, we consider 5 s for message creation time. In the 5th second, the proposed technique reaches a proportion of 0.99 than that of the existing techniques [CDRAM (0.96), GFAVR (0.86), vehicular TMS (0.92)].

**Figure 4:**
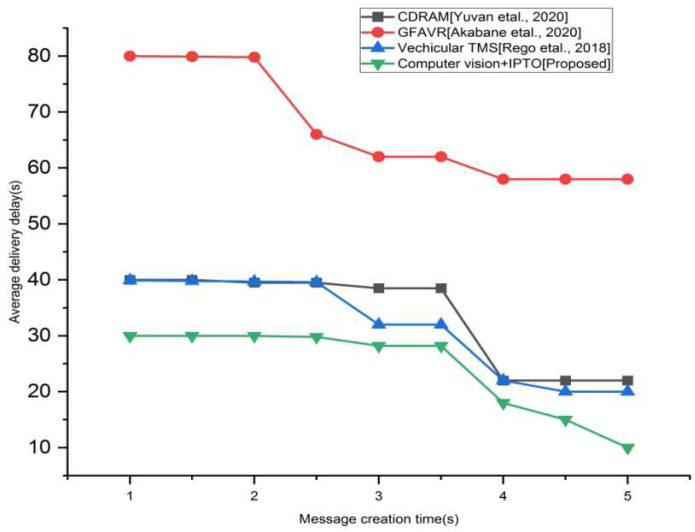
Average delivery delay vs. different message interval for the existing and proposed method.

As depicted in Figure 4, the suggested approach offers a significant advantage over conventional methodologies of average delivery delay. Here, the proposed technique has little delay than that of the existing approaches. That means that the proposed approach has a 30 s delay than the existing approaches CDRAM (40 s), GFAVR (80 s), vehicular TMS (38 s) in the first second of message creation time. In the 5th second, the proposed technique reaches a 10 s delay than that of the existing techniques [CDRAM (18 s), GFAVR (56 s), vehicular TMS (17 s)].

**Figure 5:**
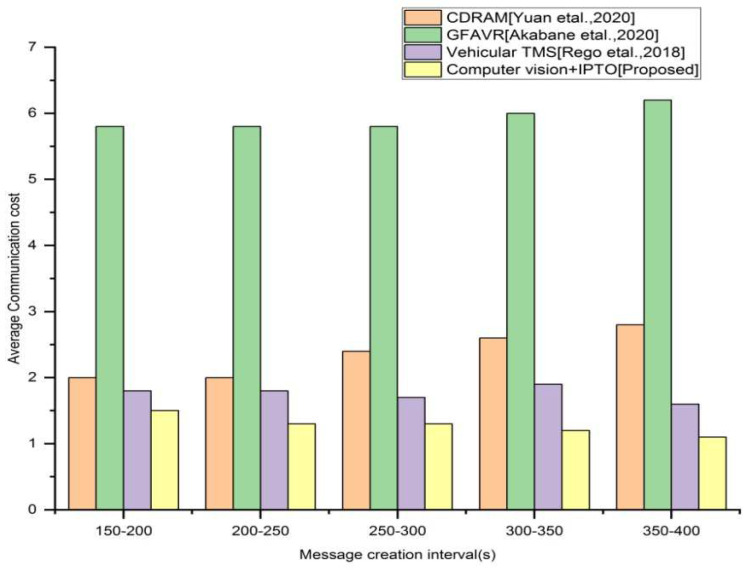
Average communication cost vs. different message interval for the existing and proposed method.

As depicted in Figure 5, the suggested system’s average communication cost is significantly lower than that of the current approaches. Here, we consider 400 s for the message creation time. The proposed technique has a minimized communication cost than that of the existing approaches in overall 400 s of the message creation time. However, in 200–250 s, the proposed approach has very little average communication cost than that in 150–200, 250–300, 300–350, and 350–400 s.

**Figure 6:**
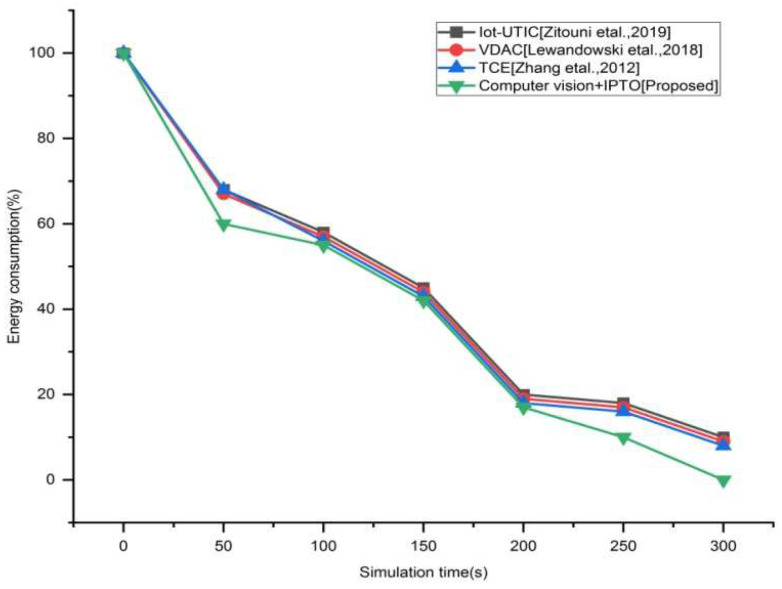
Average energy consumption vs. simulation time for the existing and proposed method.

The quantity of energy utilized by the nodes in proportion to the simulation time is referred to as energy consumption. Figure 6 shows how the suggested approach uses less energy than existing techniques such as IOT-ULC, VDAC, and TCE. Since there are a large number of vehicles that circulate on city roadways, our system uses a substantial amount of energy in 80 percent of traffic density. However, owing to the high traffic congestion rate on the roads and reduced vehicle traffic, we find that energy consumption in the network is stable since the identification statuses of our sensors have not changed.

**Figure 7:**
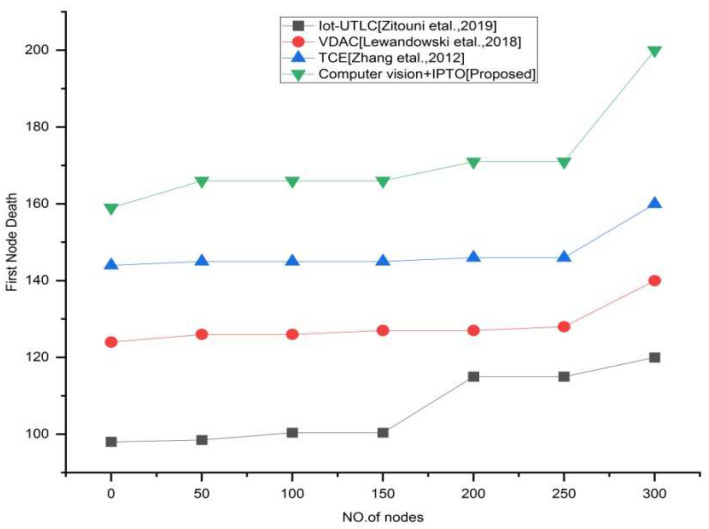
First node death vs. number of nodes for the existing and proposed method.

**Figure 8:**
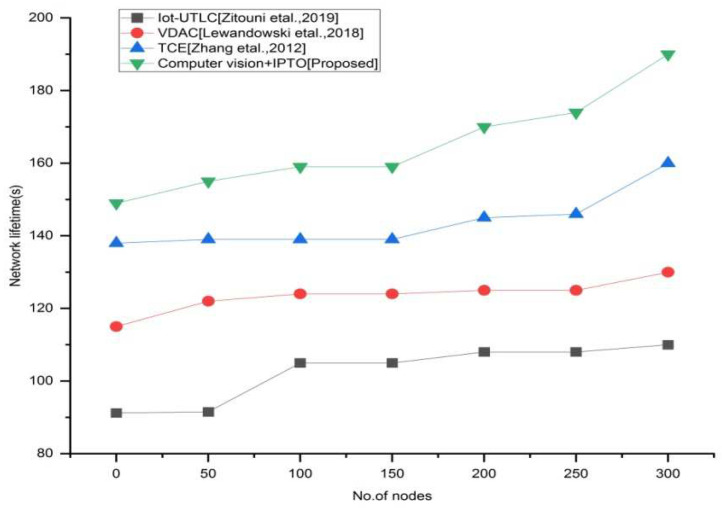
Network lifetime vs. number of nodes for the existing and proposed method.

We use two key indicators to indicate the network’s lifespan. The first is the time until the “first node dies (FND)”. Since a node dies during this time, the FND period is considered a period of network stability. The second is total network life, which is defined as the period when no more nodes are available to continue communicating; this duration is referred to as “network life (NL)”. The result shown in Figure 7 and Figure 8 relates to the network’s lifespan. We found that by deploying an efficient and effective network architecture that adjusts to changes in the density of current traffic on the roadways, our suggested system enhances the network’s lifespan, making it an intelligent and innovative system. Other current systems, on the other hand, employ basic and common methods that allow for the same unsatisfactory outcomes throughout the network’s lifespan.

**Figure 9:**
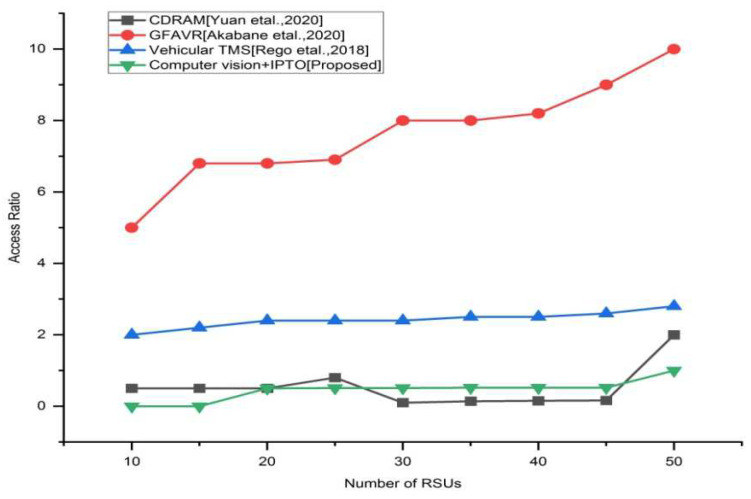
Access ratio vs. different number of RSUs for the existing and proposed method [8,25,26].

Figure 9 depicts the access ratio of the proposed approach vs. traditional methods, which reflects RSU and BS resource use. In terms of access ratio, the proposed solution is superior. Here, the proposed technique has a smaller access proportion than that of the existing approaches. That means the proposed approach has a proportion of 0.1 then the existing approaches CDRAM (0.2), GFAVR (5), and vehicular TMS (2) in 10 RSUs. In this work, we consider 50 RSUs. In 50th RSU, the proposed technique reaches a proportion of 0.5 than that of the existing techniques [CDRAM (3), GFAVR (10), Vehicular TMS (3.5)]. Finally, we prove the proposed approach is superior to the existing approaches.

## 5. Key Challenges and Future Directions

### 5.1. Challenges

The Internet of Vehicles’ quick advancement is deteriorating numerous barriers in the development of smart cities and urban planning, but there are still numerous key challenges to overcome such as security, reliability, interoperability, real-time communication, multi-model sensing, and heterogeneity.

- **Security and Privacy:** Applying different techniques need to handle secure communications. Moreover, the wireless nature of wireless sensor transmission poses a great risk to cyber-attacks than fixed-line infrastructure. Interference allows for easy eavesdropping or denial of wireless transmission. This makes it a prime target for hackers, with the significant potential catastrophic harm. Furthermore, sensors can be used without the need for safeguards or observation. This raises the danger of attackers exploiting the mobile nodes and gaining access to sensitive data or credentials to launch an attack via remote access. Sensors can potentially keep confidential material on the privacy of their clients.
- **Reliability:** Data dependability is critical for urban transportation operation and assessment, however, due to its minimal exposure and restricted capability of sensing wisps, as well as the lossy nature of transmissions, it became difficult while employing WSN. As a result, any proposed WSN architecture and communication protocols should incorporate providing reliable edge connections.
- **Interoperability:** Traffic control is an inter-optimizing issue that necessitates the improvement of a variety of weighted parameters such as gas emissions, number of stops, and traffic noise levels, among others. This multiplicity of feasible solutions necessitates a multiplicity of sensing devices, which is one of WSN’s primary design goals.
- **Real-time Communication:** Various approaches involve real-time data collecting to make in-the-moment choices. A typical scenario is traffic signal control, in which variable crossroads planning is dependent on real-time traffic distribution.
- **Multi-model Sensing or Heterogeneity:** Due to the fast advancement of wireless technologies and many sensing devices, suggested systems must consider equipment diversity to assure compatibility and flexibility.

### 5.2. Future Directions

Any Remote wireless sensor-based transportation management system must be able to tackle the aforementioned issues. WSN innovation presently meets certain standards, making it a popular contender for practical Intelligent Transport Systems (ITS) applications. The vulnerability of WSN and its subsystems should be considered while developing traffic control services depending on WSN. The communication systems, in particular, has to be secured enough to mitigate damage while also being dependable and fault—tolerance capable of providing services stability in the face of failure. WSN’s multisensory aspect is strong since it enables the improved traffic flow multi-objective dilemma to be fed with crucial data in real-time, allowing the optimal transportation planning decisions to be taken at the moment. Blockchain and Artificial Intelligence are emerging technologies and scientists are working closely to integrate these technologies in smart cities since blockchains is distributed technology with enhanced security and cost-effective technology [30,31] while artificial intelligence-based technology (e.g., machine learning, deep learning [32,33]) are intelligent technologies. These technologies should be explored in future research to improve the performance of IoV in terms of traffic management systems.

## 6. Conclusions

Traffic congestion is a continuous issue across the world, posing both economic and social issues. Any city’s ability to compete depends on its ability to maintain smooth traffic conditions. Hence, we presented a novel computer vision-based traffic management system that incorporates a wireless sensor network and a visual analytics framework. We present an improved phase timing optimization approach to optimize traffic data. The suggested approach also has the advantage of allowing visual analysis of traffic dependency between roadways in urban networks. It aids in determining the geographic consequences of a specific activity in a given area. The whole experiment was carried out under the MATLAB environment. Communication cost, access ratio, energy consumption, network lifetime, delivery ratio, and delay are the parameters used to evaluate the proposed system with conventional methods. The proposed approach of this study is low in cost, high in instability, and does not require large-scale building or installation work, as opposed to the usual way of monitoring vehicle traffic. Furthermore, the future system should be able to provide other traffic data such as occupancy, queue length, and categorization type. Furthermore, the proposed approach may be adopted in other areas where double parking and bustling roadside activities are a problem.

## Data Availability

Not applicable.
